# Conceptualising the Commercial Determinants of Health Using a Power Lens: A Review and Synthesis of Existing Frameworks

**DOI:** 10.34172/ijhpm.2021.05

**Published:** 2021-01-25

**Authors:** Benjamin Wood, Phillip Baker, Gary Sacks

**Affiliations:** ^1^Global Obesity Centre, Deakin University, Geelong, VIC, Australia.; ^2^Institute for Physical Activity and Nutrition, Deakin University, Geelong, VIC, Australia.

**Keywords:** Corporate Power, Corporate Influence, Commercial Determinants of Health, Corporate Strategy

## Abstract

**Background:** There is increasing recognition that power imbalances that favour corporations, especially those active in unhealthy commodity industries, over other actors are central to the ways in which corporations influence population health. However, existing frameworks for analysing corporate strategies and practices that impact on health do not incorporate concepts of power in consistent ways. This paper aimed to review the ways in which corporate power has been incorporated into such frameworks, and to propose a revised framing of the commercial determinants of health (CDoH) that makes concepts of power explicit.

**Methods:** We conducted a narrative review of frameworks that identify corporate strategies and practices and explain how these influence population health. Content analysis was conducted to identify explicit references to different qualities of power – its origins, nature, and manifestations.

**Results:** Twenty-two frameworks were identified, five of which used theories of power. A wide range of contexts that shape, and are shaped by corporate power were discussed, as were a diversity of corporate, social and ecological outcomes. A variety of material and ideational sources of power was also covered. We proposed an integrated ‘Corporate Power and Health’ framework to inform analysis of the CDoH, organised around key questions on power set out by Foucault. The proposed framework draws from a number of well-established corporate power theories and synthesises key features of existing CDoH frameworks.

**Conclusion:** Public health advocates, researchers and policy-makers would likely be better placed to understand and address the CDoH by engaging with theories of power to a greater extent, and by explicitly incorporating concepts of corporate power in analyses of how the deployment of corporate strategies and practices influence population health.

## Background

 For decades, the public health community has recognised the need to understand and address the ways in which corporate actors influence population health,^1-7^ particularly those active in health-harming industries such as the tobacco, processed food, alcohol, firearm, motor vehicle, gambling, and pharmaceutical industries.^4,7,8^ In particular, within the past decade there has been a large emphasis on the role of corporations in driving the rising incidence of non-communicable diseases (NCDs).^5,7,9^ This focus is unsurprising considering that NCDs result in more than two-thirds of deaths and disability worldwide.^10,11^ Importantly, the increased attention to the ways in which the for-profit private sector can shape social circumstances to the detriment of population health has represented a paradigm shift within the field of public health, with focus moving away from a framework of social determinism that emphasises weakness and disadvantage towards one that instead scrutinises the role of power and politics in shaping health.^5,7,12,13^ Stemming from this recent shift in thinking, the field of *corporate* and *commercial determinants of health *(CDoH) emerged.^7,14,15^

 There is increasing awareness that at the heart of the CDoH lies the subject of corporate power.^13,16-18^ It has been argued that, in contexts in which there is limited constraint on corporate power, dominant corporations have managed to influence many different aspects of society, from the macrostructural components (ie, policy and regulatory spaces) to the moulding of individual behaviours and consumption patterns, in order to protect and pursue their interests.^8,17,19^ Furthermore, many in the public health community have argued that key structural changes within the global political economy – including the promotion of neoliberal thinking across different social spheres, the internationalisation and liberalisation of trade and capital, and the deregulation of markets – have led to shifts in power that favour corporations over public health interests.^6,13,16,17^ To date, however, corporate power has not been a mainstream focus of the public health community, and the role that corporate actors play in influencing population health has likely been understated.^20^ Many existing public health frameworks that do aim to explain the ways in which corporations influence health do not fully engage with theories of corporate power, and thus may be limited in their comprehensiveness and explanatory power.

 Power is a complex and highly contested subject with multiple definitions and conceptualisations across many different academic disciplines. As a starting point, this paper draws from Foucault’s supposition that power has three distinct qualities: its basic nature, its origins, and its manifestations.^21^ Our rationale for beginning here is that, although the conceptualisation of each of these qualities may vary substantially, the majority of well-established theories of *corporate* power do not appear to deviate from these qualities. In regard to public health, there are a number of well-established theories of the nature and origins of corporate power that are well placed to strengthen understanding of how corporations influence health. We recognise that the third quality of corporate power, its manifestations, is an inherent component of all CDoH research.

 To inform our understanding of the basic nature of corporate power, we draw from Fuchs’ *three forms of corporate power* framework.^22,23^ This framework, in a similar fashion to many political science approaches to power, emphasises the relationship between actors, structures, and ideas by stating that corporate power can exist in three different but interconnected forms – instrumental, structural and discursive. Instrumental power can be considered the ability of corporate actors to directly influence other actors, generally referring to influencing political decision-making via actions such as lobbying.^24^ Structural power refers to the ability of corporations to predetermine processes of decision-making and non-decision-making through the shaping of the options that are, or are perceived to be, available to other actors.^24^ Lastly, discursive power is the power of corporations to influence public opinion and political processes through the shaping of values, norms and ideas.^24^ Since its introduction, Fuchs’ model has been widely used to explore the expression and effects of corporate power in a number of different areas, including public health,^25-27^ international political economy,^28^ food and agricultural systems,^29^ and environmental sustainability.^24^ Fuchs’ framework was developed out of a perceived need, particularly within the International Relations literature, to adapt and apply existing political power frameworks to global corporations.^22,23,28^ It is important to note that Fuchs’ framework draws heavily from Lukes’ *three faces of power* framework,^30,31^ one of the most recognised theoretic models of power. Lukes’ *three faces of power *describes three different expressions of power. Drawing from the work of Robert Dahl in the 1950s and 1960s, Lukes recognises that decision-making power, its most visible form, is expressed through the ability of one actor to directly influence the actions of other actors in decision-making spaces.^32,33^ Lukes also describes non-decision-making as a hidden form of power, wherein actors have the ability to set the agenda and thereby make certain topics off-limits for discussion in decision-making spaces and public forums.^30,34^ The third form of power is ideological in nature, wherein power in its most diffuse and invisible form can be expressed via shaping the *perceived* options of other actors through the creation and legitimation of norms and ideas.^30,35^

 As Fuchs et al note, and consistent with Foucault’s qualities of power, it is also useful to look beyond the ways in which corporate power can be expressed by exploring the sources of its expression.^24^ In this respect, we draw from work by Fuchs and Glaab, which expands on Fuchs’ earlier three forms of corporate power model.^36^ This work recognises that scholars have usually agreed in making the distinction between *material* sources of corporate power (ie, access to and control of technological, natural or economic resources and assets) and *ideational* sources of corporate power (ie, social constructs such as norms, values, ideas and knowledge).^24,36^ Importantly, the ability of corporate actors to draw from ideational sources of power to influence discourse, an expression of discursive power, is linked to their perceived legitimacy.^36,37^ Both *material *and *ideational* sources of power are deeply interlinked with the nature and manifestations of corporate power. In many cases, making a distinction between the three power qualities can be very difficult, and they may also vary across time and space. For instance, the ideational sources of power of a corporation today, especially those linked with neoliberal thinking such as freedom from government control, were likely constructed in part by earlier discursive strategies deployed as part of a broader pro-corporate movement.^38^ As an another example, the generation of substantial profits (both within and across geographic markets), a manifestation of corporate power, perpetuates the considerable material sources a powerful corporation has as its disposal (which might then be deployed within a different space).^39^

 In light of the aforementioned gaps, this paper had two primary aims. First, we aimed to review existing frameworks for analysing the influence of strategies and practices used by corporations active in health-harming industries on population health to examine whether theories of power have been explicitly integrated, and to determine if and how they have discussed the different qualities of corporate power. Second, we aimed to synthesise relevant theoretical models of corporate power with the identified conceptual frameworks to create an integrated corporate power and health framework.

## Methods

 This paper involved a narrative review of existing frameworks designed to explain the ways in which the strategies and practices of corporations active in health-harming industries influence health. Identified documents were qualitatively examined using content analysis that was framed according to an integrated power framework described below. Adopting a framework synthesis approach, the analysed qualitative data from existing frameworks were synthesised to create an integrated corporate power and health framework.^40,41^

###  Search Strategy 

 We conducted a scoping search on Medline, Scopus, Web of Science and Business Source Complete for documents published from any date up until the 30/9/2020, using the terms: (‘corporate determinants’ or ‘commercial determinants’ or ‘corporate power’ or ‘corporate influence’) AND health AND (framework or model or concept or approach). This resulted in the identification of 495 papers, 84 of which were duplicates. The bibliographies of retrieved papers were also searched, as well as the first ten pages of Google Scholar using the same search terms. The list was supplemented with the authors’ own knowledge of existing frameworks.

###  Literature Selection and Data Extraction

 Titles and abstracts (or table of contents and/or executive summaries if abstracts were not available) were screened for all search results. The process of selecting framework documents was guided by Kickbusch and colleagues’ commonly used definition of the CDoH, ie, ‘*strategies and approaches used by the private sector to promote products and choices that are detrimental to health.*’^5^ Specifically, we attempted to identify framework documents that explicitly endeavoured to explain the ways in which the use of corporate strategies and practices influence population health outcomes. We excluded general discussion documents that did not have an overarching framework. Additionally, framework documents that only focused on a single corporate strategy or practice (eg, marketing) were excluded. Full texts were retrieved following screening and independently reviewed and tabulated by one of the authors. These initial findings were then reviewed by two authors, with discrepancies resolved via a consultation process. Data extracted included author(s), date, title, and, when available, underpinning theory or theories of power used as part of the proposed framework or its development. We sorted the identified papers in order of the year they were published and coded them according to whether the framework document (ie, the entire text) made reference to the qualities of power as described in the developed framework below.

###  Framework Analysis and Synthesis 

 We developed a framework for content analysis based on Foucault’s three qualities of power – its origins, nature and manifestations.^21^ We drew from existing corporate power frameworks, (introduced in the background section of this paper) to inform our understanding of the origins and nature of corporate power.^36,42^ As part of exploring the nature of corporate power, we also considered the underlying contextual factors and dynamics of institutional arrangements (ie, the structured set of rules that frame the interaction between different actors) that shape, and are shaped by, corporate power.^42^ Importantly, these ‘sets of rules’ that structure interactions between actors are not static in nature. They are subject to being shaped by changes in underlying contexts and paradigms (eg, the emergence of neoliberalism as the dominant global economic paradigm), as well as being directly shaped by powerful corporate actors (eg, shifts in governance towards private modes).^6,23,42^ We drew from the business management and critical political economy literature to broadly categorise the institutional arrangements that enable or constrain corporate power based on the context in which they are embedded.^43,44^ We also recognised that institutions can be formal (eg, laws, contracts, form of government) or informal (eg, traditions, customs, beliefs, values).^42,45,46^ Additionally, institutions can exist at the national, supranational or subnational level, or be more diffuse and transversal in nature (eg, technological and ideological institutions).^42,45,47^ Finally, we proposed an integrated corporate power and health framework that was developed by integrating the framework for analysis (described above) with a synthesis of analysed framework documents.

## Results

###  Review Findings 

 We identified 22 documents with frameworks or models designed to explain how the strategies and approaches used by private actors influence population health outcomes (*refer to [title of supplementary document] for the corresponding PRISMA flow chart for the review*)^4-9,13,16,17,48-52^ (see Table). Collectively, the frameworks covered a wide range of social, cultural, political, economic, ideological and ecological manifestations of the expression of corporate power that influence population health and well-being. Of the 22 frameworks, five explicitly used a theory of power to help link the effects of corporate power with the ways in which it is exercised.^16,17,51,53,54^ Most of the frameworks described different institutional arrangements or social structures that shape, and are shaped by, corporate power, with the majority focusing on the role of policy-making and regulatory institutions (at both national and supranational levels) in enabling or constraining corporate power.^4,5,8,9,16,17,48,51^ In combination, a diverse range of important material and ideational sources of corporate power were covered.

**Table:** An Overview of the 22 Identified Conceptual Frameworks and Models for Examining the Ways in Which Corporations Influence Health, and Their Key Features From a Power Perspective

**Author(s) and Year of Publication**	**Title of Framework Document**	**Explicit Reference to Power**	**Theory of Power Integrated Into the Framework**	**Key Features**
Saloojee and Dagli, 2000	Tobacco industry tactics for resisting public policy on health	No	No	One of the first studies in the public health literature to aggregate strategies and tactics used by the tobacco industry to influence public health policy
Spitzer, 2005	A systemic approach to occupational and environmental health	Yes	No	Strong focus on the social structures that enable corporate power and reinforce population harm
Freudenberg and Galea, 2008	The impact of corporate practices on health: implications for health policy	No	No	The use of case studies from different industries to highlight how corporate practices influence health
Jahiel, 2008	Corporation-induced diseases, upstream epidemiologic surveillance, and urban health	Yes	No	Uses an upstream multilevel epidemiologic approach to explain the flow of corporate power through social environments
Holden and Lee, 2009	Corporate power and social policy: the political economy of the transnational tobacco companies	Yes	Yes – Farnsworth and Holden’s corporate power framework^55^	The use of power theory to examine the corporate involvement of transnational tobacco companies in social policy
Wiist, 2010	Tactics of the Corporation (In: The Bottom Line or Public Health)	Yes	No	Strong focus on treating the corporation as a distal, macro-level social structure
Freudenberg, 2012	The manufacture of lifestyle: The role of corporations in unhealthy living	Yes	No	Explores how corporations influence lifestyles that shape patterns of ill-health
Moodie et al, 2013	Profits and pandemics: prevention of harmful effects of tobacco, alcohol, and ultra-processed food and drink industries	Yes	No	The use of extensive unhealthy product sales data across numerous countries to support claims
Millar, 2013	The corporate determinants of health: how big business affects our health, and the need for government action	Yes	No	Coined the phrase ‘corporate determinants of health’
Mialon et al, 2015	A proposed approach to systematically identify and monitor the corporate political activity of the food industry with respect to public health using publicly available information	No	No	Adapted existing tobacco industry-related corporate political activity frameworks to the food industry
Kickbusch et al, 2016	The commercial determinants of health	Yes	No	Defined and popularised the ‘CDoH’
Baum et al, 2016	Assessing the health impact of transnational corporations: its importance and a framework	Yes	No	Focus on both positive and negative effects of corporate actors; description of outcomes of corporate practices in broad range of social and environmental contexts
Knai et al, 2016	Systems thinking as a framework for analysing the commercial determinants of health	Yes	No	The use of a systems thinking framework in order to analyse a complex issue from multiple perspectives
Ulucanlar et al, 2016	The policy dystopia model: an interpretive analysis of tobacco industry political activity	Yes	No	Provides a comprehensive approach to understanding the discursive and instrumental political strategies used by tobacco corporations that influence public health policy
Madureira-Lima and Galea, 2018	Corporate practices and health: a framework and mechanisms	Yes	Yes – Lukes’ three faces of power ^30,31^	The use of power theory to explain how different corporate practices can translate into expressions of power, depending on the context in which the practice is deployed
Thorn, 2018	Addressing power and politics through action on the commercial determinants of health	Yes	No	Focus on political science literature critical of the pluralist view that business corporations are subordinate to the political process and an elected government
McKee and Stuckler, 2018	Revisiting the corporate and commercial determinants of health	Yes	Yes – VeneKlasen and Miller’s power framework^56^	The use of power theory to how the expression of corporate power is becoming increasingly hidden and invisible
Rochford et al, 2019	Reframing the impact of business on health: the interface of corporate, commercial, political and social determinants of health	Yes	No	Encourages a stronger focus on the positive aspects of the influence of business on health; explores the internal processes of business
Brown, 2019	Legislative capture: a critical consideration in the commercial determinants of public health	Yes	Yes – Flyvberg’s phronetic research methodology^57^	Draws from phronetic research methodology to explore the role of power and values in legislative capture by corporations
Eastmore et al, 2020	Non-market strategy as a framework for exploring commercial involvement in health policy: a primer	No	No	The use of a non-market strategy perspective from business literature to explore commercial involvement in health policy
Walls et al, 2020	Advancing alcohol research in low-income and middle-income countries: a global environment framework	Yes	No	Use of a novel conceptualisation of the alcohol environment to explore how alcohol corporations influence local alcohol use
Jamieson et al, 2020	Oral health inequalities and the corporate determinants of health: a commentary	Yes	Yes – Lukes’ three faces of power ^30,31^	Explores how corporate power influences oral health by integrating Lukes’ three faces of power with Kickbusch and colleagues’ channels of corporate power

Abbreviation: CDoH, commercial determinants of health.

###  The Origins of Corporate Power

 In relation to the origins of corporate power, most framework documents did not explicitly reference a specific power theory, but typically did imply that corporations draw from important material and ideational sources of power. For instance, most of the documents mentioned that many important corporate practices – eg, the use of extensive marketing and public relations, lobbying – require substantial amounts of money. In terms of other material sources, it was mentioned that certain corporations have access to key research and development-related funding and assets, such as state subsidies and access to basic research conducted in government facilities, which can facilitate the generation of intangible assets via intellectual property channels.^7^

 Most of the framework documents also discussed a number of important ideational sources of power, and in particular, the use of ‘*social constructs such as ideas, identities, values and norms and drawing on symbolic structures of meaning.*’^24^ A number of the documents discussed examples where corporate actors have drawn from the social constructs of democracy and individual freedom. For example, it was described how corporations often portray government attempts to regulate markets as an infringement of personal choice and freedom of speech.^7,16^ Knai et al referred to how The Coca-Cola Company has historically used symbolism that embodied ‘*Western open-market values, freedom, happiness, youthful culture, and democracy*’ to promote its products.^49,58^ For example, Coca-Cola distributed their soft drink brand to millions of East Germans only a few hours after the Berlin Wall was torn down in 1989, with more than two million people reportedly drinking* ‘a toast to freedom with a Coke*’ in the following week.^49,59^ Corporate actors were also stated to draw from elements of youth, Black, or feminist movements to promote their products.^52,60^

 Another important source of ideational power discussed was that of controlling and withholding information. This recognises that what is generally perceived as fact or truth can be created and shaped by the communication strategies of different corporate actors and their decisions to disseminate or withhold information.^36^ For example, it was mentioned that certain corporations active in unhealthy commodity industries withhold important information (eg, the addictive nature of substances used in products), and, thus, control what knowledge is available to society.^9,60-62^

###  The Nature of Corporate Power – the Different Expressions of Corporate Power

 Five of the framework documents explicitly integrated theories of power into their conceptualisations in order to explain how the use of strategies and practices by corporations can be understood as expressions of corporate power vis-à-vis other actors, structures and/or ideas. Two frameworks linked a range of corporate strategies and practices to Lukes’ *three faces of power*. Another drew from a related power framework introduced by VeneKlasen and Miller.^16^ The framework document of Holden and Kelly applied the conceptual framework developed by Farnsworth and Holden to analyse the corporate power of transnational tobacco corporations.^51^ In their power theoretic model, Farnsworth and Holden distinguish agency power from structural power, with corporate agency power referring to taking explicit action (similar to Fuchs’ understanding of instrumental power), and corporate structural power referring to situations in which governments are constrained to act in ways that protect or promote corporate interests without corporations having to resort to explicit action (similar to Fuchs’ understanding of structural and discursive power).^55^ Lastly, Brown applied Flyvbjerg’s phronetic planning methodology to explore the role of corporate power in legislative capture.^54,57^ The remaining 17 framework documents described corporate strategies and corresponding practices without explicitly making a theoretical link between the use of corporate strategy and the expression of corporate power.

 Using Fuchs’ three forms of corporate power, we grouped the corporate strategies collectively described according to instrumental, structural or discursive expressions of power.^22,23^ Instrumental power, which tends to draw from actor-specific material resources, can be considered the ability to directly influence other actors.^24^ Lobbying was the instrumental power strategy most frequently described, with corporate actors seeking out to directly influence political decision-makers.^4,5,7-9,16,17,50,54,55,60,63-65^ Other instrumental power strategies mentioned included the provision of political campaign donations,^4,8,17,50,55,60,65^ the provision of gifts to key decision-makers,^55^ the use (or threat of use) of litigation to deter action that could bring attention to unhealthy products or practices,^8,17,63-66^ intimidating opposition via practices such as physical or online harassment,^8,65^ and applying direct pressure on international trade and investment negotiations, either directly or through representatives from their government.^8,17,55^

 An examination of the structural power of corporations draws attention to the material conditions that influence actors’ choices, and how material structures can shape the real and perceived options of other actors.^24^ In this respect, corporations can deploy strategies that shape the social structures in which actors interact, notwithstanding the range of external factors outside of direct corporate control that can also shape these very same social structures. Examples of structural power included the power bestowed upon dominant corporations from industry-friendly modes of governance (eg, voluntary self-regulation, public-private partnerships),^9,49^ the integration into and consolidation across global value chains,^5,50,52,55^ the acquisition of ownership in media organisations (thereby setting the agenda for these media),^16^ gaining access to key decision-making spaces though practices such as ‘revolving doors,’^16,17^ and limiting the choice and availability of purchasing options for consumers.^9,52^ A number of framework documents also described that a number of large transnational corporations (TNCs) have relied on their structural power relative to national governments to threaten the shifting of jobs and capital abroad if undesirable regulations were to be implemented,^16,55^ Such corporate structural power relative to governments is strengthened in cases where corporations collude to form coalitions, such as trade associations.^64^

 Finally, a focus on discursive power – the most subtle and diffuse form of power – exposes how interests, problems and solutions can already be defined before decision-making processes commence.^24,28^ Discursive power draws on both ideational sources (eg, the use of ideas, beliefs, values and norms; consumer and political legitimacy) and material sources (eg, substantial amounts of money are required to create and shape knowledge).^22,24,28^ Some of the discursive power strategies used by corporations considered were the use of extensive marketing and public relations campaigns (including integrating marketing into online spaces, such as social media, to target younger audiences),^4,5,8,9,16,17,49,55,60,63,64^ shaping the public health-related evidence base to protect sales of unhealthy products and practices,^4,8,9,16,17,49,50,55,65^ the use of corporate social responsibility initiatives to build legitimacy and deflect attention from tarnished reputations,^5,8,63^ shaping the policy process by constructing arguments about the economic importance of their operations,^65,66^ and co-opting grassroots organisations and movements in an attempt to both confer legitimacy to corporate claims and to deflect criticism.^8,17,55,63-65^

###  The Nature of Corporate Power – the Contexts in Which Corporate Power Is Distributed 

 In understanding the different expressions of corporate power, it is important to explore the contexts in which corporate power exists and is distributed, as well as the underlying dynamics and paradigms that shape these contexts. The review identified two deeply interlinked aspects of context analysis. The first aspect explores how corporations *shape* these underlying contexts, dynamics and paradigms, which is, in effect, an extension of analysing their structural and discursive power. The second aspect explores how corporate power is *shaped by* underlying social contexts, dynamics and paradigms.

 In relation to underlying dynamics and paradigms that shape the contexts in which corporate power is expressed, a number of framework documents paid attention to the ways in which neoliberalism and market fundamentalism in particular have led national market economies and the global political economy to acquiesce to corporate power.^13^ Discussions on some of the ways in which this has partly occurred included describing how neoliberal and market fundamentalist thinking have favoured the implementation of policies and legislations that have promoted market deregulation, the liberalisation and internationalisation of trade and finance, the privatisation of public services, the reduction of the size of government, minimisation of government intervention (including the weakening of competition policy), and the strengthening of private property rights.^5,7-9,13,16,48,54,55,67^

 Within the political economic context, the majority of the framework documents made reference to the increasing ability of corporate actors to shape political and regulatory institutions at both the national and supranational levels.^4,5,8,9,16,17,48,52,55,60,67^ Specifically, reference was made to the shift in governance towards public-private partnerships within the United Nations system, as well as across many national governments, as an important institutional dynamic that has increased the structural power of already powerful corporations.^9,22,23^ Baum et al described how binding trade agreements have reduced the capacity of national governments to regulate the activities of certain corporations in order to protect population health within their jurisdictions.^48^ It was also described that markets and their regulatory institutions have been structured to allow corporations to externalise considerable social and environmental costs, as well as to consolidate dominant positions in increasingly concentrated markets.^6,8^ Furthermore, corporations have been able to integrate into, and consolidate control over, value chains spanning across an increasing numbers of countries due to institutional arrangements that facilitate the internationalisation of trade, capital and production.^5^ The deregulation of capital mobility and the increase in corporate ‘transnationalisation’ have also enabled corporations to pursue financial strategies, such as tax minimisation.^8^

 A number of frameworks referred to key structural factors and dynamics within the legal context, such as the use of courts and dispute settlement mechanisms by corporate actors to counter and deter opposition, as well as the special rights that have been bestowed upon corporations (eg, rights as ‘persons’ under the law, limited liability for shareholders, the right to initiate contracts).^4,6,8,16,17,54^ The extra-legal context was also considered, encompassing the institutional arrangements and contextual factors that enable corporations, especially tobacco corporation, to avoid paying tax and facilitate illegal trade.^8,17,55,64,66^

 A range of factors were discussed in relation to the sociocultural and ideological contexts that shape, and are shaped by, corporate power. This included using the mass media (an industry that in many contexts has become increasingly reliant on business advertising for funding) to reinforce corporate values and ideas; the corporate provision of curriculum, school funding, and teacher training to shape educational material; the shaping of research through corporate capture of research institutions and think tanks; and the promotion of social environments and cultures that encourage individualism and increased consumption.^4,6,7,9,16,17,49,52,55,60,67^ Walls et al also provided the example of how the alcohol industry exploited and exacerbated social inequalities in South Africa, referring to a system in which alcohol was used as an informal payment system for farm labour and how this contributed to harmful consumption in disadvantaged populations.^52,68^

 Lastly, the technological and related regulatory context was depicted as encouraging the continual commercialisation of novel technologies, such as genetically engineered crops, without the need for corporations to fully consider the potential health and environmental risks that they may pose.^6^

###  The Manifestations of Corporate Power

 In the frameworks identified, the outcomes of the expression of corporate power were mostly framed from both a corporate perspective and a public health perspective. More than half of the framework documents made reference to the perceived ultimate goal of corporations in the current era: the maximisation of profits and shareholder value.^4-9,49,54,60^

 Beyond the exploration of specific public health outcomes (such as mortality and morbidity directly related to tobacco use, the misuse of alcohol, and unhealthy diets), a number of framework documents also looked at how the use of corporate strategies and practices can result in a diverse range of social and ecological outcomes – both negative and positive – that either directly or indirectly affect population health and well-being. A number of the outcomes of corporate power discussed were structural changes to the different institutional arrangements discussed in the previous section. For example, a few noted that, within the political-legal context, an outcome of the expression of corporate power has been policy, regulatory and legislative capture, the situation in which decisions over policies, regulations and legislations are directed away from the public interest and towards corporate interests.^17,49,54,55^ Similarly, Millar described potential effects of corporate power as being political instability, the diversion of government money spending away from essential public services in order to cover the costs of their externalities, and the inhibition of economic growth.^7^ Baum et al alluded to some of the benefits that can arise from the operations of TNCs, particularly in relation to investment in emerging national economies, such as improvements in employment opportunities, working conditions, education, infrastructure and healthcare provision.^48^ On the other hand, Baum et al also mentioned that TNCs can lead to a wide range of adverse outcomes, such as the creation of dangerous working conditions, the provision of inadequate pay, the dislocation of traditional communities, and the negative impacts on local businesses.^48^ Importantly, a number of framework documents revealed a range of ecological outcomes that influence population health which can result from the operations of powerful corporations, such as environmental pollution, the reduction of water quality, the clearing of land, the consumption of substantial amounts of energy, the production of large amounts of waste, and significant contributions to greenhouse gas emissions.^6,48,52^

###  Proposed Framework for Linking Corporate Power and Population Health Outcomes

 We propose the ‘Corporate Power and Health’ framework to link corporate power and population health, organised using Foucault’s three qualities of power – its origins (ie, from where corporate power is sourced), basic nature (ie, how it is expressed and the contexts in which it exists and is distributed), and its full range of corporate, social and ecological manifestations (see Figure).^21^

**Figure:**
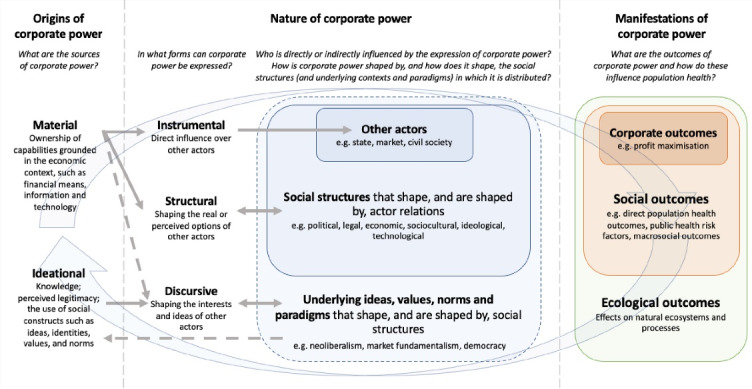
An Integrated Corporate Power Framework to Inform Analysis of the CDoH. Abbreviation: CDoH, commercial determinants of health. Adapted from Foucault’s three qualities of power,^21^ Fuchs’ three forms of power,^22,23^ Fuch and Glaab’s sources of corporate power,^36^ and Madureira-Lima and Galea’s corporate practices and health framework.^17^

 The first component of the proposed framework (‘origins of corporate power’) encourages an examination into the sources of corporate power. This component draws from Fuchs and Glaab’s sources of corporate power framework to describe the origins of corporate power as being either material or ideational in nature.^36^ While an examination of obvious material sources of power (eg, money) is clearly important, the proposed framework encourages exploration of different material sources of power (eg, ownership of intangible assets) and ideational sources of power (eg, using arguments based on concepts of ‘democracy’ and ‘individual freedom’ to market products in diverse geographic settings, and to frame debates against the use of government regulation).

 The second component of the proposed framework (‘nature of corporate power’) seeks to examine the forms in which corporate power is expressed, as well as the extent to which social structures shape, and are shaped by, corporate power. This component adopts Fuchs’ *three forms of corporate power* model.^22,23^ The different contexts in which corporate power is distributed were categorised as being: (1) political; (2) legal (and extra-legal); (3) economic; (4) sociocultural and ideological, and (5) technological. The distinction between different contexts draws from work that has grouped institutional arrangements (or social structures) based on the character of the social system in which they are embedded.^8,17,43,44,46^ Within the political context, different political institutional arrangements, such as the various organs of government (eg, legislative branch) and government policies (eg, trade, industry and competition policy), should be considered.^43^ Institutional arrangements within the legal and extra-legal context include, for instance, those relevant to contract law, property rights, tax minimisation, and rule enforcement.^17,46^ The organisation of markets and value chains, and the structure of competition, are examples of important economic institutional arrangements.^43^ Within the sociocultural and ideological context, institutional arrangements such as political ideology, higher education systems, and systems of religious belief need to be taken into account.^43^ Lastly, technological institutional arrangements include those that support or lead efforts to advance technology (eg, corporate research and development, universities) and importantly, encompass the systems and processes that allow technological innovation to be commercialised.^69^

 In reality, the different contexts that are distinguished in the proposed framework are deeply interconnected and overlap. For instance, international trade is an economic activity that is increasingly organised by global value chains, whereas international trade agreements are largely a political instrument.^70,71^ Nevertheless, separate analyses of the characteristics of particular contexts help to identify important mediators of the expression of corporate power. Not shown in the presented framework is that institutions can exist at the national, supranational or subnational level, or be more diffuse and transversal in nature (eg, technological and ideological institutions).^42,45,47^ Accordingly, analyses should consider that power relations between corporations and other actors occur at different levels.

 Importantly, the proposed framework encourages recognition of the underlying ideas, norms, values and paradigms that shape, and are shaped by, the structures embedded within different social systems. This, in particular, speaks to the diffuse and invisible nature of corporate discursive power, as well as the role of paradigms such as neoliberalism and market fundamentalism in shaping a range of social structures in a manner that enables corporate power.

 Finally, the third component of the proposed framework (‘manifestations of corporate power’) focuses on the outcomes of corporate power. In considering the effects of corporate power, outcomes have been grouped as corporate outcomes, social outcomes and ecological outcomes. This categorisation was influenced by the range of outcomes mentioned in the identified frameworks.^6,8,48,50^ The particular outcome measures and indicators to examine in relation to each category of outcomes would be determined based on the particular subject and context. Corporate outcomes can include indicators that explore the firm-specific gains obtained from the expression of corporate power, such as an estimation of the costs externalised by the firm (ie, a valuation of the estimated reduction in societal welfare not borne by the firm) and metrics related to market power (eg, market concentration, price-cost mark ups).^72^ Corporate outcomes could also encompass potentially positive effects on societal welfare, such as the number of employees and their working conditions (eg, wages and entitlements, work culture, occupational health standards, gender and race representation). Social outcomes encompass population health outcomes, such as direct health outcomes related to occupational standards and exposures, as well as more distal outcomes related to increases in population exposure to risk factors (eg, tobacco, unhealthy diets, alcohol, gambling). Furthermore, social outcomes also refer to changes to the ‘macrosocial’ determinants of health, such as political outcomes (eg, policy capture leading to the implementation of policies, eg, excessive corporate tax breaks and bailouts, that divert resources from essential public services towards corporations), economic outcomes (eg, the collapse of local business due to their inability to compete with larger transnational firms), and sociocultural outcomes (eg, the shaping of evidence as a result of increases in industry-funded research).^73^ Effectively, in many cases, these macrosocial outcomes reflect changes in the social structures in which corporate power exists and is distributed. Ecological outcomes may include issues such as the extent to which a firm contributes to carbon emissions, air and water pollution, waste (including waste related to product packaging), and land clearing.

## Discussion

 The study reviewed existing frameworks designed to explain how the strategies and tactics of corporate actors active in health-harming industries can influence population health, focusing on the ways in which theories of corporate power were explicitly integrated into their conceptualisations. Five out of the 22 identified frameworks explicitly used theories of power to support their conceptualisations of the ways in which corporate power can be expressed and subsequently affect population health. In this respect, we recognise the frameworks of Madureira-Lima & Galea and Jamieson et al that integrated Lukes’ three faces of power, as well as different ‘channels’ or ‘vehicles’ of power, into their conceptualisations; the work of McKee and Stuckler that included VeneKlasen and Miller’s power framework; the framework of Lee and Holden that integrated Farnworth and Holden’s corporate power model into its conceptualisation; and the research of Brown that used Flyvberg’s phronetic research methodology to explicitly consider the role of corporate power in legislative capture.^57^

 Building on the work of existing CDoH frameworks, we proposed an integrated ‘Corporate Power and Health’ framework. The main purpose of the proposed framework is to support corporate strategy analysis by encouraging public health researchers to consider the use of corporate strategies as, first and foremost, expressions of corporate power. In this respect, the framework acts largely as a heuristic device that conceptualises how the different qualities of corporate power interact and intersect. The proposed framework expands on existing CDoH frameworks on two counts. First, it explicitly considers the origins of corporate power and how these interact and interconnect with the different expressions of corporate power. While a similar approach has been applied to work in other fields, such as environmental sustainability,^24,74^ our framework has been specifically tailored for a public health audience. Second, the framework synthesises a broad range of outcomes relevant to public health that result from corporate power, grouping them into corporate, social and ecological outcomes.

 The proposed framework serves as a complement to existing typological frameworks that describe the political strategies of corporations in specific sectors, such as tobacco,^66,75^ alcohol,^76^ and processed food.^77^ Furthermore, the proposed framework is well-placed to support analyses of other defining features of the CDoH, such as the underlying structural contexts and paradigms that facilitate corporate power, and the potential countervailing policy actions that could be implemented in order to protect and promote public health.^27,78-80^

 A strength of the proposed framework is that it draws from well-established corporate power theories. These theories have been organised around the key questions set out by Foucault to inform a comprehensive examination into power. To assist in understanding the nature of corporate power, we have used Fuchs’ *three forms of corporate* power model, which has previously been applied within the public health field,^25-27^ as well as in the fields of international political economy^28^ and the political economy of food systems.^24,29^ As a pertinent example, in a recent public health study (which fell outside of the search period for this paper), Milsom et al adapted the *three forms of corporate power* model to explore the different forms and mechanisms of power that are active in trade and health policy spaces, and, in particular, how certain corporations prevent the implementation of policy actions on NCDs.^27^ The framework presented by Milsom et al highlighted how the different mechanisms of power are active in different spaces (closed, open, invited, claimed) and at different levels (global, national, local), based on Gaventa’s power cube.^47^ Nevertheless, to the best of our knowledge, the framework presented in the current paper is the first within the public health literature to apply corporate power theoretic models to examine the interconnections among the origins, nature and manifestations of corporate power. Of course, there are a number of other corporate power theories and models that exist in the wider scholarly literature, many of which emphasise the relationship between actors, structures, and ideas. One notable example is that of Farnsworth and Holden, which has already been briefly introduced in this paper. Another notable example is Levy and Newell’s Neo-Gramscian approach to exploring the material, discursive and organisational dimensions of corporate power, in part to understand how corporate actors can effect change within complex social systems.^81,82^ This approach has been used to examine the power of automobile and fossil fuel corporations within the realm of environmental governance.^79^ In other research, such as work that has explored the political economy of finance capital, quantitative analyses of market capitalisation and firm profitability trends have been used to identify and monitor long term trends in corporate power, drawing from *Capital as Power* theory that posits capital itself does not symbolise labour or utility, but *power*.^83,84^ Such a quantitative approach draws parallels with the work from the traditional and new empirical industrial organization literature, which has typically focussed on identifying market and broader economic aspects of corporate power.^85,86^ Notwithstanding the limited understanding of the utility and applicability of different corporate theories and approaches to public health research, we argue that proposed framework serves as a useful point of departure.

 This paper has a number of important limitations. First, we did not search for documents that focused on specific corporate strategies. Although the paper did not aim to systematically review corporate strategies, we nevertheless recognise that relevant work, such as that by Miller and Harkins that examined lobbying and public relations from a public health perspective, was not included in the study.^87^ Second, our use of search terms such as ‘commercial determinants,’ ‘corporate determinants’ and ‘corporate influence’ are potentially quite specific to public health; therefore, we recognise that we may have missed relevant literature in other fields that used different terms. Similarly, we used only four databases for our literature search, although in combination, these databases cover a diverse range of literature, including public health, social sciences, business, arts and humanities, and science and technology. Third, the proposed framework has not yet been applied in practice, and specific guidance for tailoring the framework for application in particular contexts have not yet been developed. An example of a future research avenue could involve examining in depth how the qualities of corporate power interact and interconnect in different contexts relevant to public health. In addition, future public health work could look at how the framework could be applied to explore the existence and distribution of power in different types of decision-making spaces outside of policy-making spaces (ie, the use of integrated power strategies).^82,88^ In this respect, Appelbaum’s in-depth examination of the power of pharmaceutical corporations to control distribution channels through consensus building across interest and institutional barriers illustrates the advantages of considering both market and non-market dimensions of corporate strategy in understanding corporate power.^89^

## Conclusion

 In many cases, public health advocates, researchers and policy-makers are likely to be better placed to understand and address the CDoH by increasing their engagement with theories of corporate power. Insofar as corporate strategy can be understood as an expression of corporate power, we argue that a power-explicit approach based on the different qualities of power – its origins, nature, and manifestations – encourages a deeper examination into the mechanisms by which the use of strategies and practices by corporations, especially those in health-harming industries, influence population health. A power-explicit approach could also serve to help identify a broader range of countervailing actions designed to curb corporate power that protect and promote public health.

## Ethical issues

 Not applicable.

## Competing interests

 Authors declare that they have no competing interests.

## Authors’ contributions

 Conception and design: BW, PB, and GS; Acquisition of data: BW; Analysis and interpretation of data: BW, PB, and GS; Drafting of the manuscript: BW; Critical revision of the manuscript for important intellectual content: GS, PB; Supervision: GS.
